# First Experience of Keeping Pogonophorans (Annelida: Siboglinidae) in Laboratory Conditions

**DOI:** 10.1134/S001249662205009X

**Published:** 2022-10-27

**Authors:** A. A. Prudkovsky, N. P. Karaseva, M. N. Rimskaya-Korsakova, T. P. Pimenov, N. N. Rimskaya-Korsakova, V. V. Malakhov

**Affiliations:** 1 Moscow State University, Moscow, Russia; 2 Shirshov Institute of Oceanology, Moscow, Russia

**Keywords:** frenulate pogonophorans, Siboglinidae, *Siboglinum fiordicum*, cultivation of marine invertebrates

## Abstract

The article describes the parameters of maintaining the gutless symbiotrophic annelid *Siboglinum fiordicum* in laboratory conditions outside the marine environment for 64 days.

Siboglinidae Caullery, 1914 is a family of marine annelids, which are all devoid of a digestive tract. Their nutrition is derived from symbiotic bacteria, which inhabit a special organ known as the trophosome. Frenulate pogonophorans (the subfamily Siboglininae Caullery, 1914) are the largest group of the family and are widespread in all oceans of the globe. Frenulate pogonophorans are slender worms and live in chitin tubes embedded in soft deposits. Symbionts of certain frenulate pogonophorans are capable of oxidizing hydrogen sulfide or methane [1–5]. Frenulate pogonophorans are of great interest as potential indicators of undersea accumulations of hydrocarbons [6–8]. It is therefore of importance to study the ecological physiology of frenulate pogonophorans and, in particular, to determine the concentrations of gases (primarily, hydrogen sulfide and oxygen) dissolved in capillary water and essential for maintaining pogonophorans alive. The possibility of culturing frenulate pogonophorans outside of their marine habitats for a long period of time has not been described in the literature so far. In this work, we attempted to culture frenulate pogonophorans in a specially designed marine aquarium in laboratory conditions and to determine the culture parameters.

*Siboglinum fiordicum* Webb, 1963 worms and mud samples were collected with a Van Veen grab sampler (0.25 m^2^) from a depth of 33–35 m near the Øpsesundet Strait (60°33′38″ N, 5°0′20″ E) in the North Sea from February 7 to February 15, 2022. Alive pogonophorans were placed in 50-mL tubes and transported in 2-L insulated flasks (Thermos, United States) with cooling agents. The worms were delivered to Moscow February 18, 2022 and placed in a specially designed aquarium at the Invertebrate Zoology Department (Moscow State University) February 22, 2022.

Bottom water samples to be tested for dissolved oxygen concentration and pH were collected with a Ruttner water sampler (KC Denmark A/S) in the the Øpsesundet Strait. Oxygen was measured by the Winkler method [9]; the temperature and salinity were measured with a SD204 CTD profiler (SAIV A/S, Norway). Hydrogen sulfide in interstitial sediment water (collected at 4 and 7 cm over the bottom surface) was measured by photometry with Methylene Blue [10]. Similar measurements of dissolved hydrogen sulfide in interstitial sediment water were performed with samples collected in the aquarium at 2–7 cm over the bottom after culturing worms for 2 months.

Our experimental device was based on a 140-L aquarium (80 × 50 × 35 cm). Water was prepared using a Geizer-prestizh 2 reverse osmosis system (Russia) with a ULP1812/2012 membrane (Vontron, China) and a Spectrum SRDI Ion Exchange Resins (United Kingdom). Marine water with a salinity of 33‰ was prepared using Coral Pro salt (Red Sea, Israel). Fine-pore filter foam sheets were placed in two sections of the aquarium to ensure mechanical filtration and precipitation of stirred sediment. A constant temperature of 6–8°C was maintained using a Mini 600 aquarium chiller (Resun, China). The conditions were continuously monitored and the oxygen concentration and pH were regulated using a WTW DIQ/S 282 two-channel industrial controller (Xylem, United States) with a SensoLyt® SEA pH electrode (Xylem, United States) and a FDO®700 IQ SW optical sensor for dissolved oxygen (Xylem, United States). A controller of a G7J electromagnetic valve (Camozzi, Italy) turned on a carbon dioxide inflow when pH exceeded 7.6. A general scheme of the device is shown in Fig. 1. The aquarium was set 3 months before placing pogonophorans to allow its composition to stabilize.

**Fig. 1.**
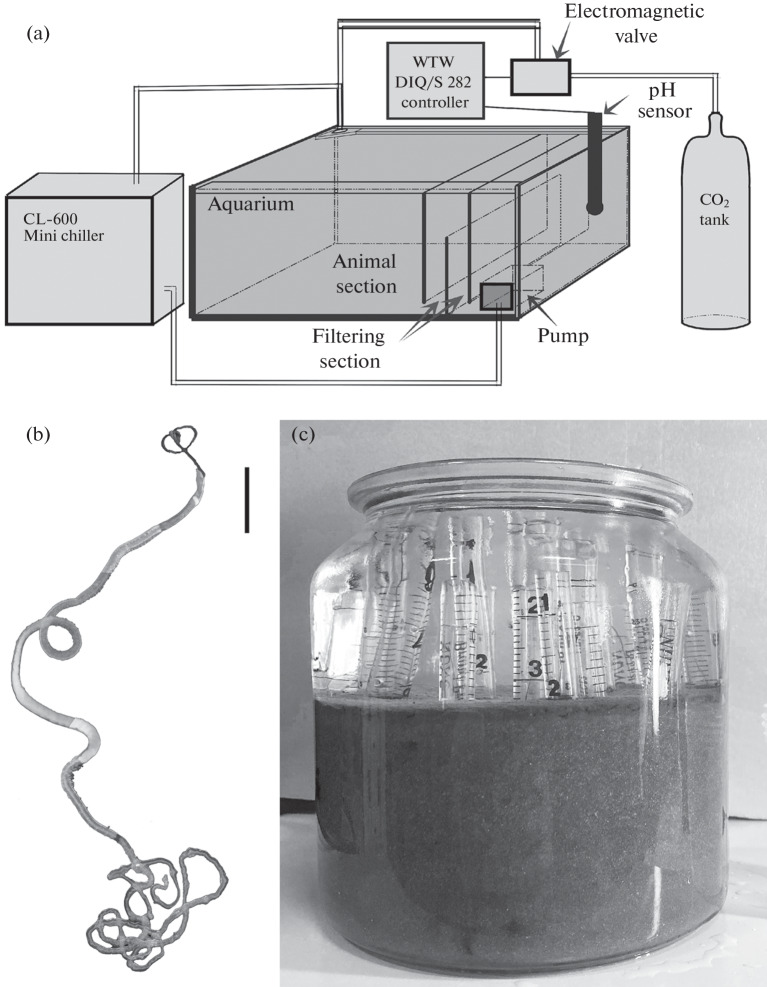
(a) Scheme of the experimental device, (b) general appearance of a *Siboglinum fiordicum* worm (bar, 1 mm), and (c) a tank with sediment used to place worms in the aquarium.

In total, 44 *S. fiordicum* worms were transferred into the aquarium*.* The worms were placed in plastic tubes with an inner diameter of 7 or 8 mm and a length of 10–15 cm. The lower end of a tube was closed with 100-µm bolting cloth, and holes of 2 mm in diameter were drilled in the walls at intervals of 1.5–2 cm. The holes ensured that water and sediment particles enter the tube. Plastic tubes with worms were placed in glass tanks (0.5–2 L), which were partly filled with sediment (Table 1, Fig. 1).

**Table 1.** Parameters of tanks placed in the aquarium and distribution of *S. fiordicum* worms at placement

	Tank 1	Tank 2	Tank 3	Tank 4
Total volume, L	0.5	0.5	1.64	2
Sediment volume, L	0.3	0.3	0.6	1.4
Sediment layer height, cm	7.5	7.5	7	8.5
Number of worms at placement	8	8	13	13
Number of worms surviving by April 26, 2022	1	0	0	2

Sediment to place the worms in the aquarium was collected together with worms in the Øpsesundet Strait and transported in 0.5- and 2-L plastic containers. The *S. fiordicum* worms were kept in the aquarium for 64 days, from February 22, 2022 (placement) to April 26, 2022 (checkup). To evaluate the worm condition, worms were extracted from their plastic tubes April 26, 2022 and examined under a stereoscopic microscope. The worm condition was evaluated by worm mobility within the chitin tube, the integrity of the anterior and posterior ends, the presence of the anterior tentacle, and the natural reddish body color. Table 2 summarizes the parameters of *S. fiordicum* culture in laboratory conditions, which were as close as possible to the environmental conditions at the site of worm collection.

**Table 2.** Comparison of the main abiotic parameters of the environment in the Øpsesundet Strait and the experimental installation. “–” means that this parameter has not been measured

Parameters	Øpsesundet Strait	Experimental installation
Bottom water temperature, °C	9.2	6–8
Bottom water salinity, ‰	33.2	33.0
Bottom water pH	7.96	~7.6
pH of pore water in sediment at a depth of 4–7 cm	7.5–7.7	–
Bottom water [O_2_], mg/L	7.71	7.65–8.5
[H_2_S] of pore water in sediment at a depth of 4–7, μmol	2.1–11.9	0.83–3.24

Three worms were found to be in good condition after being kept in the aquarium for 64 days. The results showed that frenulate pogonophores are possible to keep in laboratory conditions similar to those of their natural habitat for more than 2 months. A low survival of worms in laboratory conditions was possibly associated with a traumatic mode of worm sampling. *Siboglinum* *fiordicum* live in relatively long (up to 243 mm) and very thin (0.2–0.3 mm in diameter) tubes. The tubes were bent when sediment was collected with a grab sampler and during extraction of worms from sediment. Damage to soft tissues of worms was caused by tube bending and might remain undetected upon visual examination. More delicate procedures of worm collection and transportation should be developed to successfully culture *S. fiordicum.*
